# The need for practical insecticide-resistance guidelines to effectively inform mosquito-borne disease control programs

**DOI:** 10.7554/eLife.65655

**Published:** 2021-08-06

**Authors:** Alice Namias, Ndey Bassin Jobe, Krijn Petrus Paaijmans, Silvie Huijben

**Affiliations:** 1 Département de Biologie, Ecole Normale Supérieure, PSL Research University Paris France; 2 Institut des Sciences de l’Evolution de Montpellier (ISEM), Université de Montpellier, CNRS, IRD, EPHE Montpellier France; 3 Center for Evolution and Medicine, School of Life Sciences, Arizona State University, Life Sciences C Tempe United States; 4 The Biodesign Center for Immunotherapy, Vaccines and Virotherapy, Arizona State University, Biodesign Institute Tempe United States; 5 ISGlobal, Carrer del Rosselló Barcelona Spain; 6 Centro de Investigação em Saúde de Manhiça (CISM) Distrito da Manhiça Mozambique; Harvard School of Public Health United States; University of Geneva Switzerland

**Keywords:** Insecticide resistance, Anopheles, Aedes, vector control, surveillance, policy

## Abstract

Monitoring local mosquito populations for insecticide resistance is critical for effective vector-borne disease control. However, widely used phenotypic assays, which are designed to monitor the emergence and spread of insecticide resistance (technical resistance), do not translate well to the efficacy of vector control products to suppress mosquito numbers in the field (practical resistance). This is because standard testing conditions such as environmental conditions, exposure dose, and type of substrate differ dramatically from those experienced by mosquitoes under field conditions. In addition, field mosquitoes have considerably different physiological characteristics such as age and blood-feeding status. Beyond this, indirect impacts of insecticide resistance and/or exposure on mosquito longevity, pathogen development, host-seeking behavior, and blood-feeding success impact disease transmission. Given the limited number of active ingredients currently available and the observed discordance between resistance and disease transmission, we conclude that additional testing guidelines are needed to determine practical resistance—the efficacy of vector control tools under relevant local conditions— in order to obtain programmatic impact.

## Background

Vector-borne diseases such as dengue, Zika, leishmaniasis, Lyme disease, and malaria account for more than 700,000 deaths annually (World Health Organization, 2020b). Several mosquito-borne diseases, including dengue, Zika, chikungunya, and West Nile virus, have (re)emerged over the decade (Succo et al., 2016; Weaver, 2014), which has led to an increase in morbidity and mortality (Gould et al., 2017). To illustrate, the Zika outbreak in 2016 infected more than 130,000 people in Brazil alone (Pan American Health Organization / World Health Organization, 2017), and there are an estimated 96 million cases of dengue annually, with more than 3.9 billion people in over 128 countries at risk of contracting the disease (World Health Organization, 2020b). Moreover, malaria still kills an estimated 405,000 people annually, and half of the world’s population is thought to be at risk. Despite intensified malaria control and elimination efforts over the past two decades, it has been 5 years without significant reduction in the number of malaria cases globally. Therefore, and because of the current impact of COVID-19 pandemic on those efforts, the targets outlined in World Health Organization (WHO)’s Global Technical Strategy for Malaria 2016–2030 (World Health Organization, 2015) are unlikely to be met (Feachem et al., 2019; World Health Organization, 2020a; World Health Organization, 2020c).

With no (prophylactic) drugs and/or vaccines available to prevent or control many vector-borne infectious diseases, chemical vector control remains a cornerstone in disease control and prevention (Bhatt et al., 2015; World Health Organization, 2015). Insecticides are widely used in public health to reduce vector populations. Interventions include chemical fogging to control *Culex* ssp. (vectors of, e.g., West Nile virus and lymphatic filariasis) and *Aedes* ssp. (vectors of, e.g., dengue, yellow fever, and Zika), and indoor residual spraying (IRS) and long-lasting insecticidal nets (LLINs) for malaria control (Feachem et al., 2019). The US President’s Malaria Initiative supported IRS in over 5.7 million houses in 2018 (US Presidents’s Malaria Initiative, 2019), and over 1.8 billion pyrethroid-based bed nets have been distributed between 2010 and early 2020, of which close to 1.6 billion were distributed in sub-Saharan Africa (Roll Back Malaria, 2020). This unprecedented quantity of insecticides exerts an exceptionally strong selective pressure for resistance and, unsurprisingly, insecticide resistance to most of the WHO-approved public health insecticides has now been reported around the world (Moyes et al., 2017; Ranson et al., 2011).

For malaria vectors specifically, resistance in *Anopheles* mosquitoes to at least one class of insecticides is reported in 90% of malaria-endemic countries, and 32% of the countries have reported resistance to the four classes of insecticides (pyrethroids, carbamates, organophosphates, and organochlorines) that were recommended until 2016 (World Health Organization, 2010). Resistance to clothianidin, the latest approved active ingredient that is used in several IRS products and considered the silver bullet in insecticide-resistance management, has now been reported in Central Africa as well (Makoni, 2020). This trend of rapid increase in observed insecticide resistance following exposures is alarming as it reduces our overall chemical arsenal to control disease vectors and the efficacy of many vector control products that have been and will be deployed. To adequately manage insecticide resistance, to develop effective vector control strategies, and to understand the role of insecticide resistance in recently reduced success of malaria eradication, close monitoring of the insecticide susceptibility status in vector populations is critical.

**Figure 1.**
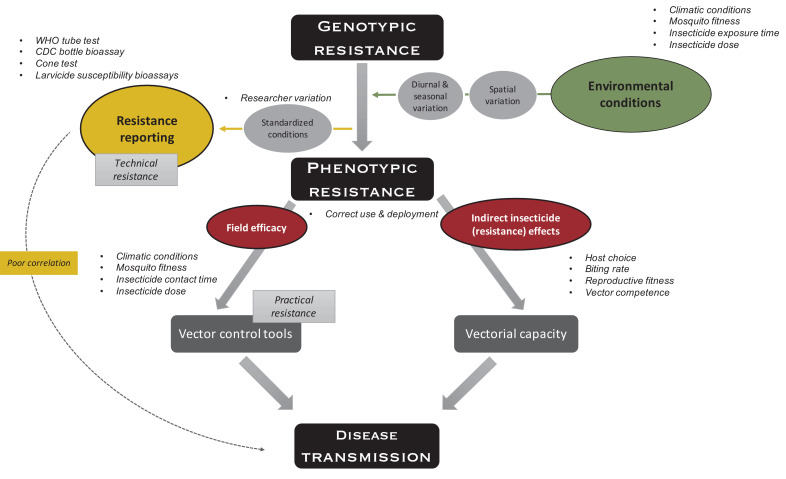
Indirect effects of environment on insecticide resistance. Environmental conditions such as climatic conditions (e.g., temperature and humidity), mosquito fitness (e.g., age, size, and feeding status), insecticide exposure time (e.g., fleeting contact vs extended exposure), and insecticide dose (depending on, e.g., time since application, washes, and insecticide half-life), which all vary over space and time, impact phenotypic resistance. To measure technical resistance, a particular phenotype (depending on the test used) is measured under standardized conditions. Variation is introduced by the researcher performing the test (e.g., variation in mosquito handling, test preparation, and mortality scoring). Phenotypic resistance translates indirectly to disease transmission: insecticide-resistance mechanisms and the insecticide itself impact the mosquitoes’ vectorial capacity (i.e., the ability of mosquito populations to transmit a specific pathogen from human to human) through host choice (e.g., frequency of human vs non-human biting), biting rate (i.e., frequency of taking a human blood meal), reproductive fitness (i.e., the ability of a mosquito to pass on her genes to subsequent generations, which is importantly impacted by survival, fertility, and ability to mate), and vector competence (i.e., the capacity of a mosquito to acquire, maintain, and transmit a parasite). In addition, phenotypic resistance indirectly translates to disease transmission through factors impacting the field efficacy of vector control tools because of aforementioned environmental conditions (i.e., practical resistance). The variation in use and deployment of the tool impacts the efficacy of the vector control tool itself, as well as vectorial capacity. Overall, these indirect effects could lead to poor correlation between standardized phenotypic tests and impact on disease transmission.

Insecticide susceptibility is typically assessed via WHO tube tests or CDC bottle bioassays for adulticides and larval bioassays for larvicides. These different tests, used around the world by researchers, non-governmental organizations (NGOs), and national and local governments, do not replicate well, are not comparable, and—maybe more importantly—do not necessarily predict if the observed resistance does actually translate to a loss in efficacy of actual vector control product and thus control failure. In this review, we (i) summarize the descriptive power of conventional insecticide-resistance assays, (ii) provide an overview of the evidence of genotype and genotype-by-environment effects on the resistance phenotype, and (iii) discuss the role of susceptibility tests in research vs programmatic surveillance programs. Whilst there are other test methodologies available that allow for more natural mosquito behaviors in response to interventions (such as the tunnel tests and experimental hut trials), we propose the development of additional scalable testing guidelines that assess the efficacy of actual vector control tools under relevant natural conditions with field-collected mosquitoes. We borrow the terms technical resistance (which we define as a heritable change in the organism that leads to reduced susceptibility in lab-controlled bioassays) and practical resistance (a heritable change in the organism that leads to mosquito control failure in field settings following correct deployment of the product) from the pesticide literature (Food and Agriculture Organization FAO, 2010) to rationalize our argument. Unlike antimicrobial resistance, where resistance directly translates into treatment failure, the effect of insecticide resistance on mosquito-borne diseases is indirect (Figure 1). For this reason, it is difficult to define vector control failure, since this could reflect failure to kill mosquitoes or failure of the intervention to have an impact on pathogen transmission. In this review, we will distinguish between the two using ‘mosquito control failure’ and ‘transmission control failure’. There is an additional important discussion to be had on how we define success and failure in each of these categories; however, this discussion is beyond the scope of this review.

## How do we currently measure resistance?

Known insecticide-resistance mechanisms in mosquitoes are target-site resistance, metabolic resistance, cuticular resistance, and behavioral resistance (see Box 1). The first three can be detected using phenotypic bioassays and/or genetic assays, whereas behavioral resistance is more difficult to detect under standardized laboratory conditions and is beyond the scope of this review.

Box 1. Insecticide-resistance mechanisms.

There are four suggested resistance mechanisms: target-site resistance, metabolic resistance, cuticular resistance, and behavioral resistance. Target-site resistance results from a point mutation in the insecticide target, preventing it to act efficiently. Target-site resistance mutations are often found in nervous system components (usually targeted by insecticides), mostly in sodium channels (targeted by dichlorodiphenyltrichloroethane (DDT) and pyrethroids), acetylcholinesterase (AchE, targeted by both organophosphates (OP) and carbamate insecticides), and in γ-aminobutyric acid (GABA) receptors. Metabolic resistance is a result of increased metabolic detoxification of insecticides through amplifications of the detoxification genes or overexpression of these same genes. It mainly involves three major detoxification gene families: cytochrome P450s, esterases, and glutathione S-transferases (GSTs) (reviewed in Liu, 2015). Cuticular resistance, associated with a decreased insecticide penetration, has been described in *Anopheles gambiae* (Yahouédo et al., 2017), *Anopheles funestus* (Wood et al., 2010), and *Culex pipiens* (Fang et al., 2015). Such a decrease in insecticide penetration is associated either with cuticle thickening or with a change in cuticle’s composition. While cuticular changes have been associated with the overexpression of several gene families, notably P450s, laccases, and ABC transporters, their exact mechanisms are still being investigated (Balabanidou et al., 2018). Behavioral resistance, also termed behavioral avoidance, leads to mosquitoes avoiding the insecticide contact. However, there is still little information available on this trait (Carrasco et al., 2019; Corbel and Guessan, 2013).

### Phenotypic resistance bioassays

The bulk of insecticide susceptibility testing is performed by testing phenotypic resistance using standardized bioassays. These insecticide susceptibility bioassays have been designed for ease of use by local mosquito surveillance authorities. The tests assess the proportion of mortality in local mosquito populations exposed to a certain diagnostic dose (predetermined using susceptible mosquito strains) under controlled laboratory conditions. Phenotypic assays can be performed on either adult mosquitoes (to test the susceptibility to adulticides) or aquatic immature mosquitoes (susceptibility to larvicides).

#### Adulticide susceptibility assays

Phenotypic adulticide susceptibility assays are predominantly performed using the CDC bottle bioassay (Brogdon and Chan, 2010) or the WHO tube test (World Health Organization, 2016b). In the CDC bottle bioassay, mosquitoes are aspirated into an insecticide-coated (or control) glass bottle. Test outcomes are knockdown after 10–90 min of exposure (Brogdon and Chan, 2010; McAllister and Scott, 2020). In WHO tube tests, mosquitoes are placed in plastic tubes lined with insecticide-treated (or control) papers. Papers with predetermined doses are prepared by a single WHO-accredited lab, although papers can be prepared by other labs that have the required equipment and consumables. Test outcomes are mortality 24 hr after a 1-hr exposure (World Health Organization, 2016b). The main advantage of the CDC bottle bioassay is the relative ease to test a variety of insecticide concentrations and the lack of need for specialized testing equipment since the bottles and insecticides are widely available. The advantage of the WHO assay compared to the CDC bottle assay is that it is less prone to individual variability, since testing equipment and treated/control papers come from a centralized source (Owusu et al., 2015). However, as a result, different mosquito species, and even genera, are tested for susceptibility using the same pre-established insecticide dose in WHO tube tests, even though CDC bottle assays have demonstrated that different mosquito species have different susceptibility profiles (McAllister and Scott, 2020).

Due to the high discordance between time-to-knockdown and mortality rates, few studies on *Anopheles* ssp. have shown similar results in mosquito susceptibility levels to insecticides between the two assays (Aïzoun et al., 2013; Vatandoost et al., 2019). This difference may result from the different assay endpoints (i.e., final read-out indicators), which is a diagnostic time of 10–90 min (depending on the insecticide tested [McAllister and Scott, 2020]) in the CDC bottle bioassay and 24 hr post-exposure in the WHO test. In other words, we assess ‘knockdown’ (i.e., the immediate effect on mosquitoes) in CDC assays, whereas we assess ‘mortality’ in WHO tube tests. CDC assays may underestimate resistance, as they will not take a so-called ‘mosquito recovery period’ into account: a phase whereby mosquitoes that are knocked down directly after the exposure recover their physiological abilities over time when no longer exposed. Such recovery is possible if the underlying resistance mechanism is metabolic and enables mosquitoes to detoxify the insecticide over time (Owusu et al., 2015). Therefore, CDC assays could fail to properly identify metabolic resistance. In addition, the mosquito’s exposure time and dose differ between CDC bottle bioassay and WHO tube test. Beyond differences in the exposure time in the assays, it has been shown that insecticide efficacy is affected by the type of surface the insecticide is applied to (Corrêa et al., 2019; Dengela et al., 2018). Insecticide pick-up may differ in the WHO assay (contact with paper surface) and the CDC assay (glass surface). Moreover, mosquitoes could avoid the insecticide-treated papers in the WHO assay if they rest on the cylindrical untreated areas at the bottom and top of the tubes. This could be a concern when testing insecticides with a repellency and/or irritating effect.

#### Larvicide susceptibility testing

Susceptibility of immature mosquitoes to larvicides can be assessed using either the WHO guidelines for larvicide testing or the so-called resistance ratio (RR) test. In both tests, third instar larvae are exposed in cups with water containing a given concentration of insecticide and mortality is recorded after 24 hr of continuous exposure (World Health Organization, 2005). The WHO larvicide test assesses the mortality of larvae to a diagnostic dose, which is twice the LC99.9, the lethal concentration killing 99.9% of a susceptible mosquito strain, of the same species (World Health Organization, 2016a). The RR test requires the simultaneous testing of mosquitoes from a known susceptible mosquito colony. The ratio between the tested strain’s LC50 and the susceptible strain’s LC50 (RR) is used as a measurement of level of resistance (World Health Organization, 2005).

The WHO larvicide assay is easier to perform as it does not require concurrent testing of a susceptible mosquito strain (which is not readily available in many study sites across the world) and only requires testing of a few dosages. But as both tests set different diagnostic thresholds forward (as seen with the adult testing procedures before), the resistance status of the same mosquito population could differ between the two tests. To our knowledge, no study has compared outcomes of these two methods. Another difficulty is that although the WHO has provided recommended diagnostic doses for insecticide susceptibility testing (Suter et al., 2017), a wide range of diagnostic doses have been used to assess resistance. To illustrate, the diagnostic dosages for temephos in *Aedes aegypti* ranged from 0.006 mg/l to 1 mg/l, a difference of more than two orders of magnitude (Supplementary file 1).

### Genetic resistance assays

Genetic resistance surveillance is regularly used in antimalarial drug resistance surveillance to determine the prevalence or frequency of drug resistance in a given area, population, or even a single patient (Nsanzabana et al., 2018; World Health Organization, 2010). If genetics is used in insecticide-resistance surveillance, the objective is typically to identify the resistance mechanism once phenotypic resistance has been detected rather than as a monitoring tool (Ishak et al., 2015; Menze et al., 2016). This is because genetic assays to detect insecticide resistance still face several challenges (Vontas and Mavridis, 2019). While the genetic base for several resistance mechanisms has been identified, genetic markers are still missing for others, and their identification is a slow and complicated process (Donnelly et al., 2016). Additionally, where genetic markers have been identified, they may not fully explain the resistance phenotype, as shown for DDT-resistant *Anopheles gambiae* in West Africa (Mitchell et al., 2014). The mismatch between single-nucleotide polymorphisms (SNPs) and resistance phenotype could be a result of several factors. First, not every gene involved is likely to have been identified (Weetman and Donnelly, 2015). Furthermore, copy number variations (CNVs) additionally confer resistance (Assogba et al., 2015; Labbé et al., 2007), and these CNVs have been demonstrated to be common in the genome and widespread in the population (Lucas et al., 2019). Such CNVs are more difficult to detect than SNPs and are therefore largely understudied (Lucas et al., 2019). Moreover, CNVs are harder to correlate with phenotype due to the duplications of both susceptible and resistance alleles. Other factors, such as ecological and climatic variability (discussed in the next section) and interactions between resistance markers, may also lead to differences in insecticide susceptibility (Edi et al., 2014; Mitchell et al., 2012). Finally, as mosquitoes are diploid, heterozygosity at the resistance loci may confer different levels in insecticide susceptibility compared to susceptibility in homozygotes (South et al., 2020). While the advantage of genetic tools is that they result in reliable replicable data, they cannot accurately assess the prevalence or frequency of phenotypic insecticide resistance at this moment in time. However, the field is rapidly improving and genetic tools may become more widely used and informative in the near future (Vontas and Mavridis, 2019).

## From controlled bioassays to field reality: spatio-temporal variation in resistance

There are standardized guidelines for each of the insecticide susceptibility tests described above, which recommend certain diagnostic dose(s), exposure time, mosquito age, resource availability, larval and adult densities, and climatic conditions. The aim of these tests is to monitor technical resistance in order to be able to compare results over time and space. However, the ecological context can considerably influence a mosquito’s susceptibility to insecticides (see Figure 1).

Increased food availability during the aquatic immature stages of various mosquito species is known to increase insecticide resistance (Grossman et al., 2018; Ong and Jaal, 2018; Owusu et al., 2017). Strikingly, the effect of high larval densities (which translates to fiercer competition for nutrients) can revert the resistance phenotype in genetically resistant larvae (Grossman et al., 2018). Similar effects are found in adult female mosquitoes, where blood-fed females are generally more resistant than unfed females (Machani et al., 2019). Moreover, a *Plasmodium falciparum* infection has been demonstrated to increase insecticide susceptibility (Alout et al., 2014b). Resistance has also been shown to change with mosquito age, with older larvae or adults being more susceptible than young ones in general (Collins et al., 2019; Ong and Jaal, 2018). Furthermore, temperature is known to affect the insecticide susceptibility phenotype across a large variety of species. Higher temperatures during the immature rearing process (Owusu et al., 2017; Polson et al., 2012) or during tests with adults (Glunt et al., 2014) have been associated with increased insecticide susceptibility, but the opposite effect has also been observed (Oliver and Brooke, 2017). The temperature-toxicity relationship may simply depend on the mosquito species tested and its existing resistance background (Glunt et al., 2018). Humidity is another climatic factor that could increase insecticide-induced mortality (Kristan et al., 2018). The fact that climatic factors affect susceptibility is no surprise, as mosquitoes are ectothermic organisms. Mosquitoes’ immune system, nervous system, and metabolic activity all depend on temperature (Kristan et al., 2018).

Given the strong influence of environmental factors on resistance phenotype and the fact that conditions such as temperature, humidity, and food availability can differ from day to day and from mosquito season to mosquito season, susceptibility test outcomes are predicted to vary over time and space. Indeed, mortality of an *Anopheles arabiensis* population after exposure to a fixed dose of permethrin and DDT went up from 33 and 36%, respectively, in the rainy season to 89 and 73% in the dry season (Abdalla et al., 2014). Variations in susceptibility have also been observed weekly: mortality rates of *Ae. aegypti* changed more than tenfold in a timespan of 2 weeks (Chen et al., 2005). Even diurnal variation in insecticide susceptibility has been observed (Balmert et al., 2014; Yang et al., 2010), which may be the result of diurnal fluctuations in the environment, the mosquito’s circadian rhythm (Rund et al., 2016), and/or daily variations in a mosquito’s immune system (Murdock et al., 2013). Apart from the temporal variation in insecticide susceptibility outcomes, strong spatial variation in resistance has been described for both *Aedes* (Deming et al., 2016) and *Anopheles* species (Foster et al., 2016; Soltani et al., 2013), and this may be a result of spatial variation of environmental factors and geographic barriers that prevent gene flow (Barnes et al., 2017). Insecticide resistance may even vary on a very fine spatial scale, down to less than 10 km, as shown for various mosquito species and genera (Deming et al., 2016; Grossman et al., 2019; Matowo et al., 2019).

Beyond variation in environmental factors, variation in insecticide exposure is also expected. The quantity of insecticides picked up by mosquitoes in laboratory bioassays will differ from the quantity picked up by mosquitoes in the field setting, due to variations in contact time and bio-availability of the active ingredients. Also, bioassays prevent the adequate assessment of excito-repellency properties an insecticide may have, possibly leading to an overestimation of mortality. To the best of our knowledge, there are no data available on the difference in insecticide pick-up between laboratory bioassays and actual interventions. It is known that insecticide availability decreases over time in both IRS applications (Sherrard-Smith et al., 2018) and LLINs (Awolola et al., 2014). Data on the actual frequency and length of insecticide exposure in the field to accurately measure the resistance selection pressure (Viana-Medeiros et al., 2018) and resistance mechanisms in local vector populations are almost non-existent (Barbosa et al., 2011). However, evidence from video tracking of mosquitoes shows fleeting contact, with the average contact time being around 1.5 min on an LLIN (Parker et al., 2015) and the median contact time of *An. arabiensis* on bendiocarb-powdered eave tube nets of 4.6 min (Sperling et al., 2017), a time period considerably less than that assayed in tube and bottle bioassays.

### Resistance phenotype and control nonfailure

Standardized phenotypic insecticide-resistance bioassays are unlikely to reflect the efficacy of vector control intervention on mosquito control in the field for the aforementioned reasons. Indeed, this gap between bioassay data and mosquito control is frequently discussed in the literature (Churcher et al., 2016; Grossman et al., 2020; Hancock et al., 2018; Ranson and Lissenden, 2016; Sherrard-Smith et al., 2018; Venter et al., 2017; Vontas and Mavridis, 2019) and even acknowledged in the WHO test procedure guidelines (World Health Organization, 2016b). This disparity between the insecticide efficacy in a lab bioassay and in a field setting may explain the fact that the strong increase in pyrethroid resistance globally (as determined by laboratory bioassays) does not always translate well to mosquito control failure, nor to transmission control failure (as reviewed in Alout et al., 2017a; Alout et al., 2017b). Since the aim of vector control is not necessarily to reduce the mosquito burden, but to reduce vector-borne disease transmission, we should not just focus on mosquito control failure but on the impact of insecticide resistance on transmission control failure. Deltamethrin LLINs have in fact been demonstrated to provide protection in Malawi despite pyrethroid resistance being common in the major mosquito vector(s) (Lindblade et al., 2015). In Kenya, schoolchildren living in areas with a high prevalence of *kdr* mutations in vector mosquitoes were less likely to be infected with malaria if they reported using pyrethroid-based LLINs (Okoyo et al., 2015). Moreover, a cohort study carried out in five countries on two continents found no significant association between pyrethroid resistance in malaria vectors and malaria prevalence or incidence (Kleinschmidt et al., 2018). However, in contrast, some other studies do suggest a link between insecticide resistance and reduced malaria control. In South Africa, switching from pyrethroids to DDT in IRS campaigns following widespread pyrethroid resistance did lead to large reductions in malaria transmission (Maharaj et al., 2005). Furthermore, a large randomized control trial in Tanzania comparing pyrethroid-only LLINs with pyrethroid-piperonyl butoxide (PBO) LLINs (with the synergist piperonyl butoxide that can partially restore pyrethroid susceptibility), in combination with or without IRS with a non-pyrethroid, showed improved malaria control with IRS or the pyrethroid-PBO LLINs, suggesting that pyrethroid resistance decreased the efficacy of the standard LLIN alone (Protopopoff et al., 2018).

In order to predict the impact of insecticide resistance on disease transmission, we need to understand which mosquito traits play a role in disease transmission and how resistance interacts with these traits (Alout et al., 2016). Vectorial capacity is the ability of mosquito populations to transmit a specific pathogen between humans (Cohuet et al., 2010). Transmission dynamics models can be used to extrapolate resistance data to make predictions on the impact of insecticide resistance on disease transmission (Briët et al., 2013; Churcher et al., 2016; Enahoro et al., 2020; Okumu et al., 2013; Sherrard-Smith et al., 2018). However, many unknowns around the impact of insecticide resistance on important parameters influencing vectorial capacity persist, such as mosquito density, blood-meal frequency, life span, pathogen development time within the mosquito, and the mosquito’s vector competence (i.e., the capacity of a mosquito to acquire, maintain, and transmit a parasite). Therefore, there is an urgent need for detailed data of randomized control trials of vector control interventions combined with contemporaneous experimental hut data to support the predictive value of these models in transmission control (Sherrard-Smith et al., 2018). For example, insecticide resistance is expected to reduce insecticide-induced mosquito mortality and thus increase the transmission potential of diseases. However, delayed mortality due to insecticide exposure has frequently been observed across different mosquito species (see for instance, Agnew et al., 2004; Martins et al., 2012) and may result in a reduced disease transmission given malaria parasites’ relatively long incubation time within the mosquito host (ranging from 7 days to several weeks, depending on temperature [Mordecai et al., 2013; Paaijmans et al., 2009] and frequency of blood-meal intake [Habtewold et al., 2021; Shaw et al., 2020]). Alarmingly though, recent observations of highly pyrethroid-resistant mosquito populations from Burkina Faso show a lack of such delayed mortality in some populations (Hughes et al., 2020).

Insecticides and insecticide resistance can influence several other vector behaviors that affect disease transmission, such as host choice and biting frequency (Alout et al., 2017b; Rivero et al., 2010; Thomas and Read, 2016). As target-site resistance is mediated through mutations of key proteins of the neural network, this type of resistance can (a) impact mating competitiveness of resistant individuals, with a *kdr* heterozygote mating advantage observed in *Anopheles coluzzii* (Platt et al., 2015) and (b) reduce biting rates, as resistant individuals may be less responsive to olfactory cues (Siegert et al., 2009). Metabolic resistance has also been associated with behavioral changes: resistant *Culex pipiens* were more predated upon than their susceptible counterparts, hence reducing mosquito densities. This is likely a side effect of esterase overproduction inducing resource depletion (Berticat et al., 2004). Vector competence can also be impacted by insecticide resistance (Rivero et al., 2010). A striking example is *Wucheria bancrofti*, a parasitic worm causing lymphatic filariasis, that can develop in susceptible mosquitoes but is unable to fully develop in highly resistant *Culex quinquefasciatus* (McCarroll and Hemingway, 2002). Such processes could especially happen in the case of metabolic resistance, whose important energetic costs could generate trade-offs between resistance and immunity and/or between resistance and parasite development. These mechanisms have been proposed among possible explanations for the increased competence of *ace-1-*resistant *Cx. quinquefasciatus* for West Nile virus (Atyame et al., 2019) and of *An. gambiae* for *P. falciparum* infection (Collins et al., 2019). In addition, glutathione S-transferase metabolic resistance has also been reported to be associated with increased *Plasmodium* infections in *Anopheles funestus* (Ndo et al., 2019; Tchouakui et al., 2019). While *kdr* resistance has been observed to reduce vector competence in the lab setting (Alout et al., 2013), contrasting results have been observed in *An. gambiae* in the field, with no association (Collins et al., 2019) or a significant positive association (Kabula et al., 2016; Ndiath et al., 2014) observed between *kdr* resistance and *P. falciparum* development. However, the majority of these studies have the limitation that they are either performed with laboratory strains of both the mosquito and the parasite or use field observations, which are confounded by the impact of insecticides on longevity, meaning older mosquitoes are more likely to be resistant but are as such also more likely to be infectious due to the long *Plasmodium* incubation period (Minetti et al., 2020). Of note is that insecticides themselves (dissociated from insecticide resistance) may also impact disease transmission. Sub-lethal exposure to insecticides may reduce host-seeking or blood-feeding success. Complex interactions between insecticide exposure and biting behaviors, giving contrasting results (increase or decrease in the host-seeking or blood-feeding success of resistant mosquitoes following exposure depending on the insecticide used), have also been observed (Diop et al., 2020; Glunt et al., 2018; Liu et al., 1986; Thiévent et al., 2019). Failure in blood feeding can be of great importance regarding malaria transmission, given that blood-meal intake could be critical for malaria development (Habtewold et al., 2021; Shaw et al., 2020). Furthermore, the exposure to insecticides negatively impacted *P. falciparum* parasites’ development in the midgut of insecticide-resistant *An. gambiae* (Alout et al., 2014a; Kristan et al., 2016) and similarly reduced the infection rate of *Plasmodium berghei* in *An. gambiae* (Hauser et al., 2020). Similarly, exposure to residual levels of insecticide to *Aedes albopictus* may lower infection rates and body titers of dengue virus (Richards et al., 2017).

### Relevance of current resistance phenotype surveillance to vector control programs

The complexities described above may explain some of the widely acknowledged discrepancies between laboratory resistance bioassay results and the observed nonfailure of vector control interventions (Churcher et al., 2016; Grossman et al., 2020; Ranson and Lissenden, 2016; Sherrard-Smith et al., 2018; Vontas and Mavridis, 2019). The WHO introduced intensity bioassays by incorporating 5x and 10x discriminating concentrations in their resistance testing guidelines, mentioning that “resistance phenotypes detected using the discriminating concentrations do not necessarily provide information in terms of efficacy failure of that insecticide in the field” (World Health Organization, 2016b). Similarly, the CDC bottle bioassay has a modified version, the Resistance Intensity Rapid Diagnostic Test (I-RDT) with higher discriminating dosages (Bagi et al., 2015; Brogdon and Chan, 2010). However, the higher discriminating concentrations, while adding some predictive benefits, still suffer from the same problems as described above (Venter et al., 2017). The missing link in this story is our understanding of the efficacy of actual vector control products in targeting local vector populations under local and natural conditions. WHO cone bioassays (World Health Organization, 2013a) with locally collected mosquitoes are an easy method to assess resistance under ambient conditions and are already frequently performed. However, these are most typically executed on the non-blood-fed F1 of field-collected mosquitoes or field-collected larvae reared to adults in the lab under room temperature or ambient lab conditions (e.g., Allossogbe et al., 2017; Bagi et al., 2015; Glunt et al., 2015; Hughes et al., 2020; Ibrahim et al., 2019; Riveron et al., 2019; Sahu et al., 2017). This approach likely fails to capture the impact of a vector control intervention on the local mosquito populations, as important factors such as mosquito age distribution, feeding status, as well as local environmental conditions that can affect insecticide toxicity, are ignored. Furthermore, the cone test does not replicate the natural contact of the mosquito with interventions, as highlighted above. There are some tests (such as WHO’s tunnel test, tent assay, cup assay, and the Mosquito Contamination Device (MCD) bottle assay) that allow for more natural mosquito behavioral responses (including transient contact and avoidance) to insecticide-treated nets, thereby assessing if the efficacy of those nets is underestimated compared to standard cone bioassays (Grossman et al., 2020; Sternberg et al., 2014; World Health Organization, 2013a). This is particularly relevant for insecticides that have a high excito-repellent effect, such as permethrin. The tunnel test uses a small experimental chamber (60×25×25 cm) between two mosquito cages. Mosquitoes are released on one side of a holed piece of net, while a suitable bait (e.g., a guinea-pig or rabbit) is placed on the other side and made available for mosquito feeding. Mortality and blood-feeding inhibition are the recorded outcomes. The MCD bottle assay consists of a 1 l bottle in which mosquitoes are released and are lured toward a piece of LLIN netting on the other side by placing a glass jar filled with hot tap water (approximately 35°C) that acts as an attractant. Immediate knockdown and mortality after 24 hr are the recorded outcomes. In addition, net efficacy tests can be performed with the Ifakara Ambient Chamber Test (I-ACT), which allows mosquitoes to interact with a human host sleeping under an actual net, mimicking the field situation (Massue et al., 2019). Its big advantage is that tests can be conducted under local natural conditions during the night when the nets are normally used and mosquitoes are active. Recorded outcomes are mortality and blood-feeding inhibition. Tunnel assays are also recommended to be conducted overnight, but other net-exposure assays are most likely performed during daytime (i.e., regular working hours), not taking the insect’s circadian rhythm into account (World Health Organization, 2013a). All the above tests are typically conducted with laboratory-reared mosquitoes. Experimental hut studies probably mimic natural conditions more closely when the efficacy of vector control interventions is evaluated, by allowing local vector populations to enter (or not) the huts (Briët et al., 2012; Massue et al., 2016), but those set-ups are costly, time-consuming, limited to few sites globally, and raise the question of how representative the artificial structures are compared to local natural houses. Meta-analyses on the predictive value of phenotypic bioassay data on the effectiveness of LLINs and IRS in terms of deterrence and blood-feeding success using hut trials may be a useful future tool for policy decision-making. However, the uncertainty in the estimates is large due to the high level of variability in the data (Churcher et al., 2016; Sherrard-Smith et al., 2018).

We, and others before us (Bagi et al., 2015; Grossman et al., 2020; Ranson and Lissenden, 2016), argue that assessments of local mosquito susceptibility against vector control products under local and natural conditions (in other words, practical resistance) are currently of utmost importance to adequately guide the decision-making process of insecticide-based interventions. Whilst technical (or standardized) resistance bioassays are valuable for tracking the emergence and spread (i.e., evolution) of insecticide resistance over space and time, they do not inform vector control programs whether the envisioned vector control product (with the same active ingredient) leads to operationally significant resistance. More worryingly, certain chemical classes may be removed from the national/local list with useful public health insecticides based on those resistance diagnostics, while actual products with the same active ingredient may still be effective (acknowledging this efficacy may differ between areas and between seasons for the variety of reasons mentioned before). With a limited insecticide arsenal available, it is important to fully understand which interventions still play a critical role in vector control.

### Developing guidelines to quantify practical resistance

To address the issue at hand, we urge policy makers and partners to develop more pragmatic and practical testing procedures to evaluate the efficacy of actual vector control tools (e.g., LLINs and IRS products). There will be no single set of guidelines to bridge the gap between technical and practical resistance, as different products have different properties. A few examples to illustrate this problem are (1) products that are known to repel mosquitoes in addition to killing them (N'Guessan et al., 2001), (2) products with slow-acting active ingredients that require delayed mortality observations (Kweka et al., 2018), and (3) non-uniform products, such as nets with deltamethrin-coated side panels but with a mixture of deltamethrin and PBO on the top panel (Kweka et al., 2017). As such, each product (or group of products with the same active ingredients) may need its own practical testing guidelines. What is clear, however, is that the body of literature highlights a need for tests to be performed under local environmental conditions (in local houses in areas where their implementation is envisioned; for IRS/LLINs: under relevant microclimatic conditions, for IRS: on relevant wall types) using local vector populations. If these mosquitoes are collected (with, e.g., non-lethal host-seeking traps [behavior relevant for LLINs] or by aspiration of mosquitoes off the walls [behavior relevant for IRS]) and used immediately in the tests, they reflect the actual local vector population we aim to kill and/or repel. One should conduct the tests at relevant times in terms of season (when tools are typically distributed, sprayed, and/or used) and time of day (when local mosquitoes are active and come in contact with the insecticide), as these times can affect their flight activity and detoxification processes (Balmert et al., 2014; Rund et al., 2016). Tests should be replicated and have an adequate number of control treatments (untreated nets or walls) due to the higher degree of variability in practical testing procedures. Two further arbitrary factors contributing to practical resistance are contact time and contact frequency (i.e., how long and how often a mosquito will contact the insecticide), which can be easily addressed by having a set of mosquitoes exposed shorter (e.g., 3 min like in the current LLIN tests) or longer (e.g., 30 min as in current residual efficacy IRS test, but note this will depend on the mode of action of the active ingredient), as well as exposing a cohort of mosquitoes multiple times during a single night to the product. However, these decisions are best guided by performing studies on actual contact times of local mosquito species with the intervention(s), similar to what has been suggested by WHO for assessing the effect of holes in LLINs on mosquito entry into a damaged net (World Health Organization, 2013b). Finally, when a product has excito-repellency properties, tests can be performed in larger cages (instead of the current WHO cones) placed against the nets, to create a refuge for mosquitoes. For larvicides, tests could be performed outside at ambient temperatures, with field-collected larvae of all stages, using original source water. Even better, but more labor intensive, is to treat several natural water bodies with larvicides following all national procedures and to add collected larvae of all stages randomly to floating meshed containers in those pools and several untreated breeding sites (serving as controls). This allows for larvae to be exposed to natural physiochemical water properties (e.g., temperature, salinity, conductivity, total dissolved solids) and food sources, but leaves them protected from predators. As larval survival is known to be dependent on larval densities (Epopa et al., 2018; Muriu et al., 2013), two or three different densities could be assessed, depending on local larval habitats. Such practical resistance data could be used to inform policy decisions, for instance, changing insecticide class when the insecticides under these natural conditions lead to a mortality below an established threshold.

While practical guidelines will inform vector control programs about the efficacy of actual vector control products, the remaining outstanding question will be how practical resistance outcomes translate to changes in disease transmission, a similar open question to technical resistance. This is especially important since practical resistance surveillance does not take the impact of resistance or insecticide exposure on mosquito transmission potential into account, which is beyond the capability of routine surveillance. We, therefore, argue for a need to collect practical resistance data to re-evaluate the association between insecticide resistance and vector-borne disease transmission, using practical resistance testing guidelines. With malaria control stagnating over the past 5 years, this new surveillance data will allow us to better understand the link between insecticide resistance, vector control efficacy, and disease epidemiology. In addition, these practical resistance data will provide vector control programs with relevant data on the efficacy of the vector control tools tailored to their local context and, therefore, the ability to make decisions on implementation strategies.

## Concluding remarks

There is an increasing amount of data on the insecticide susceptibility in mosquito vectors collected through standardized laboratory bioassays, but these data do not inform the various stakeholders about effective vector control strategies. Phenotypic resistance is the product of genotype by environment interactions, which play a crucial role in the expression of the resistance phenotype, referred to as practical resistance, with the effect not always being intuitive. While current technical resistance assays (i) are suitable to monitor the emergence and spread of local and global insecticide resistance and (ii) allow for comparisons across space and time, phenotypic resistance outcomes do not necessarily translate to the efficacy of vector control products in the field setting. It is imperative that we are more explicit about how we define ‘resistance’. Technical resistance is a heritable change in the organism that leads to reduced susceptibility in lab-controlled bioassays, whereas practical resistance is a heritable change in the organism that leads to mosquito control failure in programmatic settings following correct deployment of an insecticidal product. Therefore, for technical resistance, bioassays need to be standardized across all locations and over time. For practical resistance, such a need is absent as bioassays only need to be standardized at the local level so that the national control programs can determine whether control tools are increasing or decreasing in effectiveness over time. The different definitions of resistance as we use them here are currently not acknowledged explicitly and arguably lead to confusion for national vector control programs needing to make programmatic decisions on technical resistance data, which often do not inform them how effective actual vector control products are in the areas they are implemented for mosquito control. To support countries in making adequate vector control decisions, a different set of guidelines needs to be developed that allows for the assessment of the efficacy of actual vector control products under local environmental conditions using local vector populations. The relation between insecticide resistance and transmission control failure is harder, if not impossible, to assess with routine bioassays. Developing more pragmatic guidelines for evaluating practical resistance will allow us to better understand the link between practical resistance, mosquito control efficacy, and disease epidemiology. We do believe that technical resistance should continue to be monitored using standardized bioassays as these data inform the global spread of insecticide resistance. However, under likely scenarios of limited time, resources, and/or mosquito samples, there is an important discussion to be had with the wider community on which type of resistance monitoring should be prioritized. With limited vector control tools available, gains in malaria eradication leveling off (World Health Organization, 2020c), and arboviral diseases of both humans and domestic animals progressively becoming dominant public health problems in the world (Girard et al., 2020), we believe that giving vector control programs the tools to make evidence-based decisions tailored to their local context is pertinent to control and prevent mosquito-borne diseases.

## Additional files

Supplementary file 1. Variability in temephos resistance assays. Table shows the range of diagnostic doses used/established for temephos resistance assays in *Aedes aegypti* and methods used to establish this diagnostic dose.
