# Supplementary material for: The need for practical insecticide-resistance guidelines to effectively inform mosquito-borne disease control programs
Source: eLife. 2021 Aug 6;10:e65655. doi: 10.7554/eLife.65655 (PMC8346280; doi:10.7554/eLife.65655)
Supplement: Supplementary file 1. — Table shows the range of diagnostic doses used/established for temephos resistance assays in Aedes aegypti and methods used to establish this diagnostic dose. [file elife-65655-supp1.docx]

**Supplementary Information**

**Supplementary file 1: Range of diagnostic doses used/established for temephos resistance assays in *Aedes aegypti*, and methods used to establish this diagnostic dose.**

| Diagnostic dose | Method of diagnostic dose establishment | Reference |
| --- | --- | --- |
| 0.006 mg/L | Twice the LC99 of Rockefeller strain | ^1^ |
| 0.008 mg/L | Twice the LC99 of Rockefeller strain | ^2^ |
| 0.012 mg/L | WHO diagnostic dose | ^3^ |
|  | WHO diagnostic dose and local test | ^4^ |
|  | WHO diagnostic dose | ^5^ |
|  | WHO diagnostic dose | ^6^ |
| 0.0125 mg/L | India government recommended dose  = operational dosage | ^7,8^ |
| 0.020 mg/L | WHO diagnostic dose | ^8^ |
|  | WHO diagnostic dose | ^7^ |
|  | WHO diagnostic dose | ^9^ |
|  | WHO diagnostic dose | ^10^ |
|  | WHO diagnostic dose | ^11^ |
|  | WHO diagnostic dose | ^12^ |
|  | Twice the LC99 of a local susceptible strain | ^13^ |
| 0.03 mg/L | Susceptible strain used not mentioned  Diagnostic dose of twice the LC99 of a local strain | ^14^ |
| 0.04 mg/L | Twice the LC99 of Bora strain | ^15^ |
| 0.082 mg/L | Twice the LC99 of Bora-bora strain | ^16^ |
| 0.28 mg/L | Twice the LC99 of Rockefeller strain | ^17^ |
| 0.352 mg/L | Twice the LC99 of Rockefeller strain | ^18^ |
| 1 mg/L | Operational dosage | ^19,20^ |

**References**

1. Braga IA, Lima JBP, Da Silva Soares S, Valle D. *Aedes aegypti* resistance to temephos during 2001 in several municipalities in the states of Rio de Janeiro, Sergipe, and Alagoas, Brazil. *Mem Inst Oswaldo Cruz*. 2004;99(2):199-203. doi:10.1590/S0074-02762004000200015

2. Macoris M de LG, Andrighetti MTM, Takaku L, Glasser CM, Garbeloto VC, Bracco JE. Resistance of Aedes aegypti from the State of São Paulo, Brazil, to organophosphates insecticides. *Mem Inst Oswaldo Cruz*. 2003;98(5):703-708. doi:10.1590/S0074-02762003000500020

3. Haziqah-Rashid A, Chen CD, Lau KW, Low VL, Sofian-Azirun M, Suana IW, Harmonis H, Syahputra E, Razak A, Chin AC, Azidah AA. Monitoring insecticide resistance profiles of *Aedes aegypti* (Diptera: Culicidae) in the Sunda Islands of Indonesia based on diagnostic doses of larvicides. *J Med Entomol*. 2019;56(2):514-518. doi:10.1093/jme/tjy208

4. Pereira Lima JB, Pereira Da-Cunha M, Carneiro Da Silva R, Ribeiro Galardo AK, Da Silva Soares SD, Aparecida Braga I, Pimentel Ramos R, Valle D. Resistance of *Aedes aegypti* to organophosphates in several municipalities in the state of Rio de Janeiro and Espírito Santo, Brazil. *Am J Trop Med Hyg*. 2003;68(3):329-333. doi:10.4269/ajtmh.2003.68.329

5. Beserra EB, Fernandes CRM, de Queiroga M de FC, de Castro FP. Resistance of *Aedes aegypti* (L.) (Diptera: Culicidae) populations to organophosphates temephos in the Paraíba State, Brazil. *Neotrop Entomol*. 2007;36(2):303-307.

6. Carvalho M do SL de, Caldas ED, Degallier N, Vilarinhos P de TR, Souza LCKR de, Yoshizawa MAC, Knox MB, Oliveira C de. Susceptibility of *Aedes aegypti* larvae to the insecticide temephos in the Federal District, Brazil. *Rev Saude Publica*. 2004;38(5):623-629. doi:/S0034-89102004000500002

7. Bharati M, Saha D. Assessment of insecticide resistance in primary dengue vector, *Aedes aegypti* (Linn.) from Northern Districts of West Bengal, India. *Acta Trop*. 2018;187:78-86. doi:10.1016/j.actatropica.2018.07.004

8. Bharati M, Saha D. Multiple insecticide resistance mechanisms in primary dengue vector, *Aedes aegypti* (Linn.) from dengue endemic districts of sub-Himalayan West Bengal, India. Hwang J-S, ed. *PLoS One*. 2018;13(9):e0203207. doi:10.1371/journal.pone.0203207

9. Sivan A, Shriram AN, Sunish IP, Vidhya PT. Studies on insecticide susceptibility of *Aedes aegypti* (Linn) and *Aedes albopictus* (Skuse) vectors of dengue and chikungunya in Andaman and Nicobar Islands, India. *Parasitol Res*. 2015;114(12):4693-4702. doi:10.1007/s00436-015-4717-3

10. Shetty V, Sanil D, Shetty NJ. Inheritance pattern of temephos resistance, an organophosphate insecticide, in *Aedes aegypti* (L.). *Genet Res Int*. 2015;2015:181872. doi:10.1155/2015/181872

11. Tikar SN, Kumar A, Prasad GBKS, Prakash S. Temephos-induced resistance in *Aedes aegypti* and its cross-resistance studies to certain insecticides from India. *Parasitol Res*. 2009;105(1):57-63. doi:10.1007/s00436-009-1362-8

12. Rawlins SC. Spatial distribution of insecticide resistance in Caribbean populations of *Aedes aegypti* and its significance. *Rev Panam Salud Pública*. 1998;4(4):243-251. doi:10.1590/s1020-49891998001000004

13. Rahim J, Ahmad AH, Kassim NFA, Ahmad H, Ishak IH, Rus AC, Maimusa HA. Revised discriminating lethal doses for resistance monitoring program on aedes albopictus against temephos and malathion in Penang island, Malaysia. *J Am Mosq Control Assoc*. 2016;32(3):210-216. doi:10.2987/16-6556.1

14. Shetty V, Sanil D, Shetty NJ. Insecticide susceptibility status in three medically important species of mosquitoes, Anopheles stephensi, Aedes aegypti and Culex quinquefasciatus, from Bruhat Bengaluru Mahanagara Palike, Karnataka, India. *Pest Manag Sci*. 2013;69(2):257-267. doi:10.1002/ps.3383

15. Jirakanjanakit N, Saengtharatip S, Rongnoparut P, Duchon S, Bellec C, Yoksan S. Trend of temephos resistance in *Aedes* (Stegomyia) mosquitoes in Thailand during 2003-2005. *Environ Entomol*. 2007;36(3):506-511.

16. Leong CS, Vythilingam I, Wong ML, Wan Sulaiman WY, Lau YL. *Aedes aegypti* (Linnaeus) larvae from dengue outbreak areas in Selangor showing resistance to pyrethroids but susceptible to organophosphates. *Acta Trop*. 2018;185:115-126. doi:10.1016/j.actatropica.2018.05.008

17. Diniz MMC de SL, Henriques AD da S, Leandro R da S, Aguiar DL, Beserra EB. Resistance of *Aedes aegypti* to temephos and adaptive disadvantages. *Rev Saude Publica*. 2014;48(5):775-782. doi:10.1590/S0034-8910.2014048004649

18. Gambarra WPT, Martins WFS, Filho ML de L, de Albuquerque IMC, Apolinário OK dos S, Beserra EB. Spatial distribution and esterase activity in populations of *Aedes* (Stegomyia) *aegypti* (Linnaeus) (Diptera: Culicidae) resistant to temephos. *Rev Soc Bras Med Trop*. 2013;46(2):178-184. doi:10.1590/0037-8682-1727-2013

19. Chen CD, Nazni WA, Lee HL, Sofian-Azirun M. Weekly variation on susceptibility status of Aedes mosquitoes against temephos in Selangor, Malaysia. *Trop Biomed*. 2005;22(2):195-206.

20. Chen CD, Lee H, Chan C, Ang C, Azahari A, Lau K, Sofian-Azirun M. Laboratory bioefficacy of nine commercial formulations of temephos against larvae of *Aedes aegypti* (L.), *Aedes albopictus Skuse* and *Culex quinquefasciatus* say. *Trop Biomed*. 2009;26(3):360-365.
